# DNAJB12 mediates the causal effect of obesity on knee osteoarthritis: A mendelian randomization study

**DOI:** 10.1016/j.ocarto.2026.100877

**Published:** 2026-09-18

**Authors:** An Wang, Hongying Shan, Xi Cao, Ye Li, Xuzhao Bian, Di Zhang, Xingjie Hao, Shiquan Sun

**Affiliations:** a Center for Single-Cell Omics and Health, School of Public Health, Xi'an Jiaotong University, Xi'an, Shaanxi, 710061, P.R. China; b Collaborative Innovation Center of Endemic Diseases and Health Promotion in Silk Road Region and NHC Key Laboratory of Environment and Endemic Diseases, Xi'an Jiaotong University, Xi'an, Shaanxi, 710061, P.R. China; c Department of Epidemiology and Biostatistics, School of Public Health, Tongji Medical College, Huazhong University of Science and Technology, Wuhan, Hubei, 430030, P.R. China; d Key Laboratory of Environment and Genes Related to Diseases (Xi'an Jiaotong University), Ministry of Education, Xi'an, Shaanxi, 710061, P.R. China; e Key Laboratory for Disease Prevention and Control and Health Promotion of Shaanxi Province, Xi'an, Shaanxi, 710061, P.R. China

**Keywords:** Proteomics, Osteoarthritis, Mendelian randomization, Mediation analysis

## Abstract

**Objective:**

Obesity is a major risk factor for knee osteoarthritis (KOA), but the circulating proteins that mediate this association remain unclear. We aimed to identify obesity-regulated circulating proteins that causally influence KOA risk.

**Design:**

We conducted a Mendelian Randomization based proteome-wide association study (MR-PWAS) analysis to investigate circulating proteins mediating the effect of obesity on KOA. Using a two-step Mendelian randomization-based proteome-wide association design, we first tested genetically predicted obesity against 4907 circulating proteins from deCODE. Obesity-associated proteins were then instrumented using *cis*-pQTLs and tested for causal effects on KOA. Candidate mediators were evaluated using product-of-coefficients mediation analysis, replication datasets, tissue-specific eQTL analysis, Bayesian colocalization and multivariable MR analysis.

**Results:**

Obesity causally influenced 243 circulating proteins. Among these, DNAJB12 showed a significant causal effect on KOA risk [odds ratio (OR) 1.55, 95% confidence interval (CI) 1.24–1.95] and mediated 20.55% (95% CI 5.92–35.18%) of the obesity-to-KOA effect. Replication analyses supported the obesity–DNAJB12 and DNAJB12–KOA associations. Colocalization provided high-confidence evidence for a shared genetic signal between DNAJB12 pQTLs and KOA in the larger KOA GWAS dataset (PP·H4 = 0.9490). Multivariable MR implicated fat mass, but not fat-free mass, as the main driver of mediator protein changes and KOA risk.

**Conclusions:**

Obesity causally increases KOA risk, partially acting through obesity-regulated circulating protein DNAJB12. These findings highlight adiposity-driven pathways and fat mass reduction as potential strategies to mitigate obesity-associated KOA.

## 1 Introduction

Knee osteoarthritis (KOA) is a leading cause of chronic pain and disability worldwide, imposing a substantial and growing public health burden [1,2]. KOA is increasingly recognized as a multifactorial disease influenced by both local mechanical stress and systemic metabolic and inflammatory processes [3]. Among modifiable risk factors, obesity exhibits one of the strongest associations with KOA incidence and progression [4,5].

Obesity was traditionally viewed as a risk factor for KOA mainly through biomechanical pathways [6,7]. However, biomechanical loading alone is insufficient to fully explain the obesity-KOA relationship. Obesity is often accompanied by persistent low-grade inflammation and dysregulated immune-metabolic states [4,8,9], which may promote synovial inflammation, matrix remodeling, and cartilage degeneration. Circulating plasma protein levels can reflect an individual's metabolic and nutritional status, which are therefore highly responsive to obesity-related physiological changes [10]. As circulating proteins are distributed systemically through the bloodstream, obesity-driven alterations in their levels may exert unexpected effects on distant tissues, potentially triggering or amplifying local pathological processes [11]. Thus, plasma proteins represent accessible systemic readouts that may link adiposity to remote organ pathology, including cartilage degeneration in KOA.

In recent years, large-scale proteomics genome-wide association studies (GWAS) have identified extensive protein quantitative trait loci (pQTL), providing rich genetic information on the regulation of circulating proteins [12,13]. pQTLs are genomic regions where genetic variants influence the abundance of specific circulating proteins. Analogous to expression quantitative trait loci (eQTLs) that regulate gene transcription, pQTLs reflect the genetic architecture of the circulating proteome and, importantly, can serve as instrumental variables (IVs) in Mendelian randomization studies to infer causal effects of protein levels on disease outcomes. These resources enable systematic investigation of obesity-driven changes in the circulating proteome and their potential downstream impacts on disease risk. Thus, systematically identifying circulating proteins influenced by obesity and have causal effects on KOA can provide mechanistic insight into obesity-associated KOA and highlight key mediators along this pathway. Such mediators may further serve as actionable biomarkers and potential therapeutic targets.

MR offers a genetic epidemiology framework to strengthen causal inference by using genetic variants as IVs for modifiable exposures [14,15]. The validity of the MR analysis relies on three core instrumental variable assumptions: relevance, meaning that the selected genetic variants are robustly associated with the exposure of interest; independence, meaning that these variants are not associated with confounders of the exposure–outcome association; and exclusion restriction, meaning that the variants influence the outcome only through the exposure, rather than through alternative pathways. Large-scale pQTL studies provide an opportunity to systematically interrogate circulating proteins as intermediate phenotypes linking exposures to disease outcomes. Notably, several recent two-sample MR studies have leveraged *cis*-acting pQTLs (*cis*-pQTLs) as instruments to evaluate the causal effects of circulating proteins on OA risk [[16], [17], [18]]. However, these studies primarily focused on protein-OA relationships and did not explicitly place obesity upstream to identify proteins that mediate the obesity-associated risk. A two-step MR framework provides a pathway to link two phenotypes and intermediate factors. Previous studies have used this approach to explore the role of circulating proteins in the relationship between body mass index (BMI) and other diseases, but it has not yet been applied to investigate the mediating proteins involved in obesity-driven KOA [10,19].

In this study, we integrated large-scale obesity and KOA GWASs and pQTL of plasma proteomics within a two-step MR framework. We aimed to identify obesity-driven circulating proteins and prioritize those with causal effects on KOA, thereby providing insight into key mediators of obesity-associated KOA risk.

## 2 Methods

### 2.1 Data source

We used publicly available GWAS summary data, pQTL, and eQTL datasets. An overview of all datasets used in this study, including data sources, sample sizes, ancestry information, accession identifiers and URLs, is provided in Supplementary Table 1.

The primary exposure was genetic liability to obesity, obtained from FinnGen R12. Circulating protein data were obtained from the deCODE proteomics study [13]. The primary KOA outcome dataset was derived from a meta-analysis of UK Biobank and arcOGEN [20].

For replication, we used BMI GWAS data from UK Biobank and GIANT [21], body fat percentage (BFP) GWAS data from UK Biobank [22], independent circulating protein pQTL data from the Atherosclerosis Risk in Communities (ARIC) [23], and a larger KOA GWAS dataset (85.62% European ancestry) [24].

To evaluate transcript-level evidence, we used whole-blood eQTL summary statistics from eQTLGen [25], adipose tissue eQTL data from METSIM [26], and chondrocyte eQTL data from the United States [27]. For body composition analyses, GWAS summary statistics for body fat mass (BFM) and fat-free mass were obtained from UK Biobank.

### 2.2 Selection of instrumental variables

For diseases and anthropometric traits, single nucleotide polymorphisms (SNPs) were selected based on genome-wide significance *P* < 5 × 10^−8^. Variants in the major histocompatibility complex region were excluded, and independent SNPs were retained after LD clumping in PLINK v1.9.0 with an LD threshold of *r*^2^ < 0.001 and a 10-Mb window. Ambiguous palindromic variants were removed, and instruments with F-statistics >10 were considered sufficiently strong.

For circulating protein analyses, *cis*-pQTLs were used as genetic instruments. *Cis*-pQTLs were defined as the sites located within ±1 Mb of the transcription start site (TSS) of the protein, with MAF >0.01 and LD clumping at *r*^2^ < 0.01. To minimize horizontal pleiotropy and reduce multiplicity, we restricted instruments to *cis*-pQTLs and further excluded variants that served as instruments for more than one protein.

For tissue-specific eQTL analyses, *cis*-eQTLs were selected at *P* < 1 × 10^−5^ and processed using the same clumping and harmonization procedures.

### 2.3 Two-step mendelian randomization design

We applied a two-step Mendelian randomization framework to identify circulating proteins that may mediate the causal effect of obesity on KOA. Two-sample Mendelian randomization was performed using the inverse-variance weighted (IVW) method.

In the first step, genetic liability to obesity was used as the exposure and each of the 4907 circulating proteins measured in the deCODE proteomics study was used as the outcome. Proteins were carried forward to Step 2 only when they were Bonferroni-correction for the obesity-to-protein association (*P* < 0.05/4907) and simultaneously passed sensitivity analyses.

In the second step, obesity-associated circulating proteins identified in the first step were used as exposures, with *cis*-pQTLs serving as genetic instruments. KOA was used as the outcome. Only proteins with the same effect direction in both Step 1 and Step 2 were considered as candidate mediators. Specifically, we required that: (1) the effect of obesity on the protein in Step 1 (*β*_1_), and (2) the effect of the same protein on KOA in Step 2 (*β*_2_), produce a net indirect effect (*β*_1_ × *β*_2_) whose direction aligns with the total effect of obesity on KOA (*β*_0_).

### 2.4 Sensitivity analyses

To evaluate robustness and detect potential violations of MR assumptions, we performed the following sensitivity analyses for each significant protein. When conducting the two-sample MR, we also carried out the heterogeneity test, pleiotropy test, Steiger directionality test, and PRESSO test for outlier correction estimation simultaneously. We also conducted a reverse MR in order to avoid reverse causality. Only those results that passed the significance test, all sensitivity analyses, and did not have reverse causality were retained.

### 2.5 Mediation analysis

To assess the influence of proteins that have a causal effect on KOA in the causal effect of obesity on KOA, we used the product of coefficients method to calculate the mediation ratio of the candidate proteins.

First, we used the two-sample MR method to calculate the causal effect *β*_0_ of obesity on KOA. Subsequently, we used the effect values *β*_1_ from Step 1 regarding obesity on circulating proteins and *β*_2_ from Step 2 regarding circulating proteins on KOA. We calculated the proportion of protein mediation *β*_1_ × *β*_2_/*β*_0_ using the delta method and conducted a sensitivity test for the mediation analysis using the Sobel test.

### 2.6 SMR analysis

We applied summary-data-based Mendelian randomization (SMR) [28] test associations between genetically predicted gene expression and KOA risk. The HEIDI test was used to distinguish shared causal signals from LD, with significant SMR results and *P*_HEIDI_>0.05 considered evidence consistent with a putative causal association.

### 2.7 Colocalization analysis

We used the Bayesian colocalization analysis tool “coloc” to conduct colocalization analysis to assess whether DNAJB12 pQTL/eQTL signals and KOA shared a causal genetic variant [29]. Analyses were conducted within ±500 kb of the lead *cis*-pQTL or *cis*-eQTL variant using default coloc parameters, with PP·H4 ≥ 0.8 considered strong evidence of colocalization. We note that the coloc framework assumes a single causal variant per region, and the presence of multiple causal variants may violate this assumption, representing a limitation of the method. We used locuszoom to visualize the colocalization results [30].

### 2.8 MVMR analysis

To distinguish the independent effects of body composition components, we performed MVMR with fat mass and fat-free mass included simultaneously as exposures, accounting for their genetic correlation (*r* = 0.64) [31]. Outlier-corrected estimates were obtained using MR-PRESSO, and conditional instrument strength, pleiotropy and heterogeneity were assessed, with multivariable MR-Egger used as a sensitivity analysis.

## 3 Results

### 3.1 Study overview

The study overview is shown in Fig. 1. To elucidate biological mechanisms under obesity-associated KOA risk, we conducted a two-step MR analysis integrating obesity GWAS and large-scale plasma proteomics to identify obesity-regulated proteins and test their causal effects on KOA. We quantified mediation proportions, performed external replication studies and colocalization analyses. All data used in this study are listed in Supplementary Table 1.

**Fig. 1:**
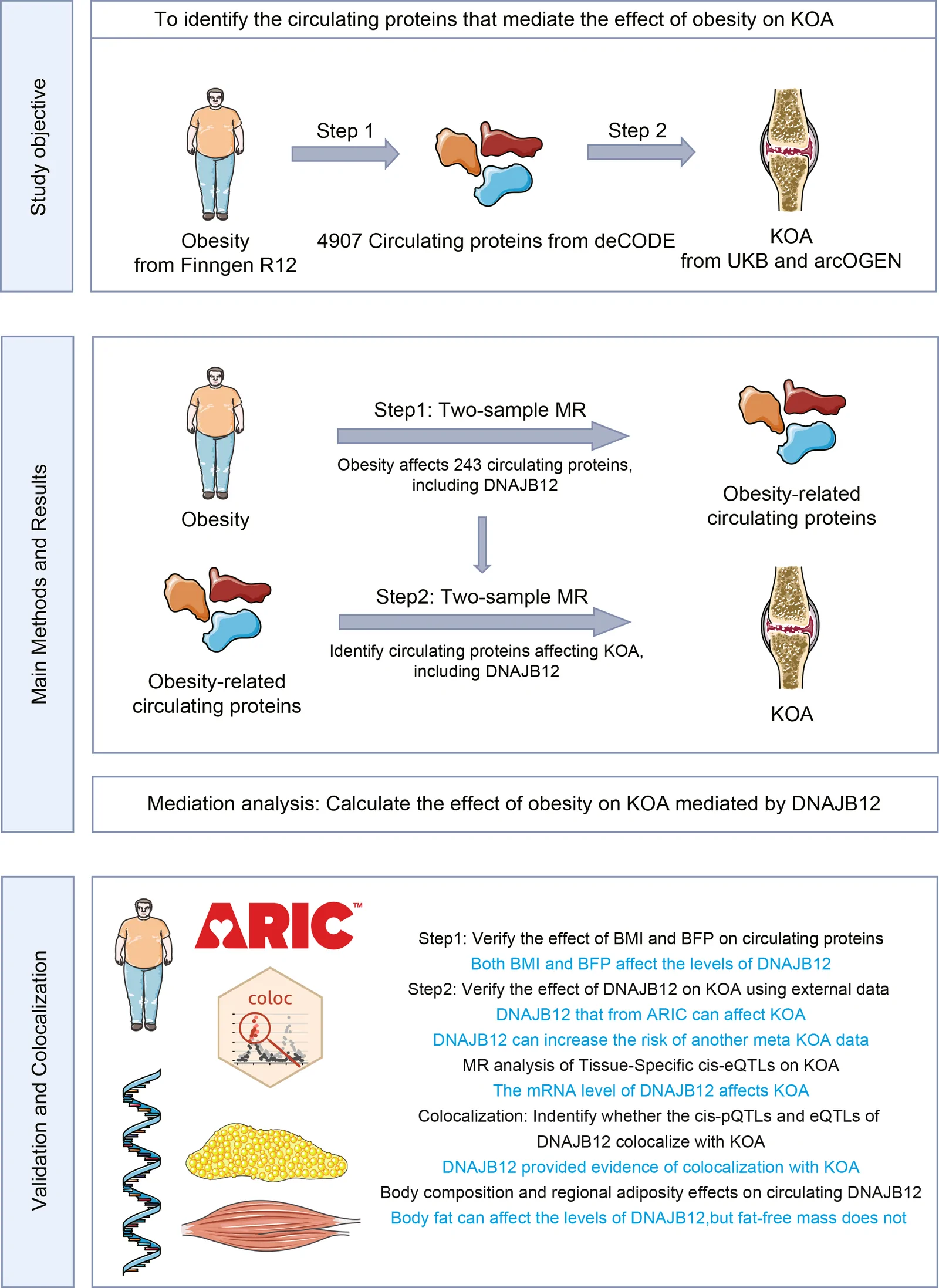
**Study overview.** Two-step MR framework integrating obesity GWAS (FinnGen R12; 500,192 participants), plasma proteomics (deCODE; 4,907 proteins; n = 35,559), and knee osteoarthritis (KOA) GWAS (UK Biobank + arcOGEN meta-analysis; 24,955 cases and 378,169 controls). Step 1 prioritizes obesity-regulated circulating proteins; Step 2 tests *cis*-pQTL-instrumented protein effects on KOA and quantifies mediation using the product-of-coefficients approach. Robustness is evaluated via sensitivity analyses, replication using independent obesity indicators and external *cis*-pQTL instruments (ARIC; n = 7,213), transcript-level evidence from whole-blood, adipose and chondrocyte eQTL (n_blood_ = 31,864, n_adipose_ = 434, n_chondrocyte_ = 101), and Bayesian colocalization (PP·H4 > 0.8). Using Multivariable MR and two-sample MR to locate the key components of body composition (All the image materials are from https://smart.servier.com).

### 3.2 MR step 1: causal effect of obesity on circulating proteins

To identify circulating proteins that are causally influenced by obesity, we conducted a two-sample MR analysis using obesity as the exposure and 4,907 circulating proteins [13] as outcomes (Fig. 2A). We obtained 68 IVs for obesity. A total of 257 proteins showed significant (*P* < 0.05/4,907 = 1 × 10^−5^) causal associations with obesity (Fig. 2B). Among them, leptin (LEP), a well-established adiposity-related hormone, showed the strongest obesity-associated increase (*β*_OB-to-LEP_ = 0.2696, *P* = 2.6385 × 10^−26^).

**Fig. 2:**
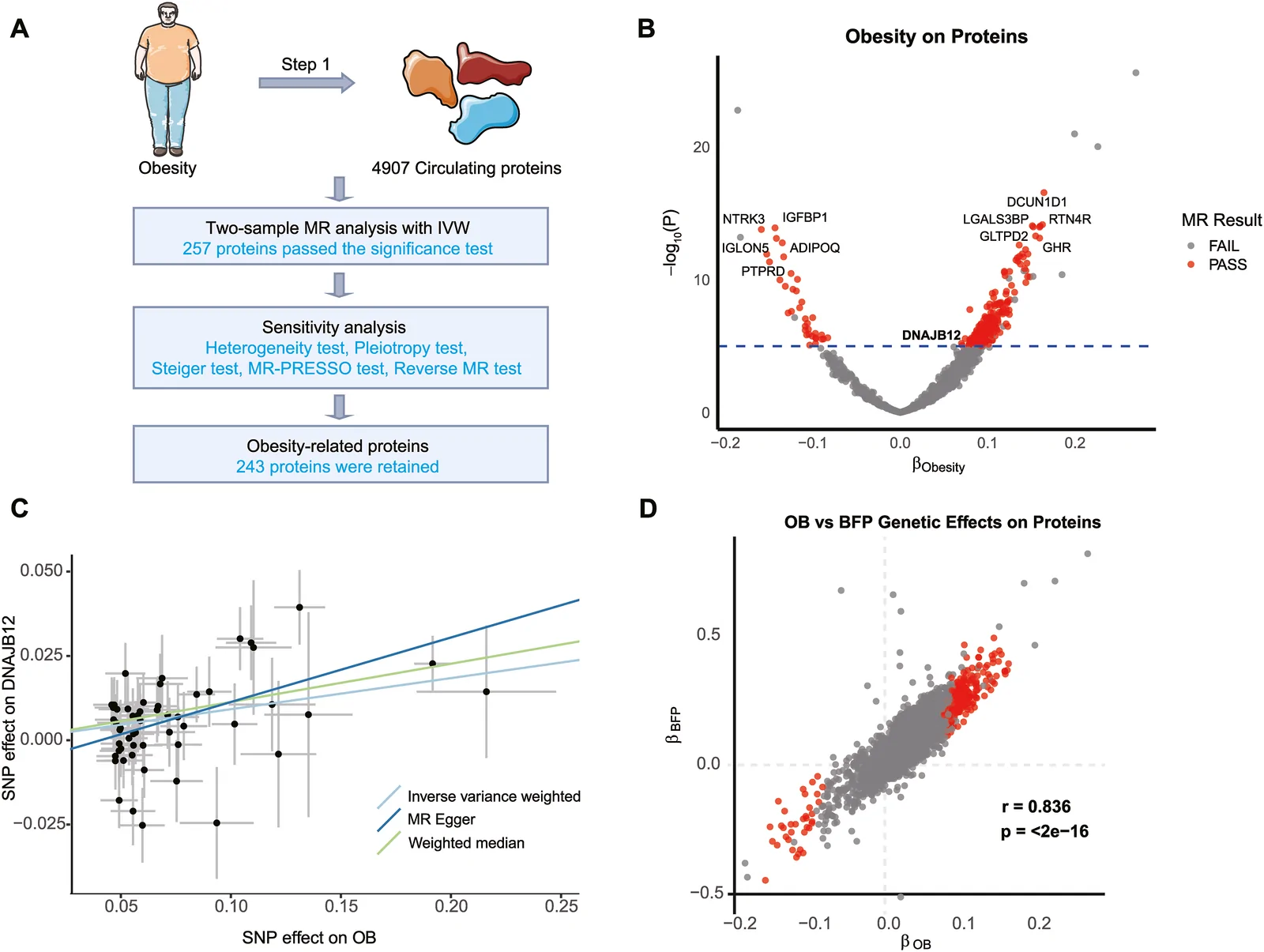
**Proteome-wide MR identifies obesity-regulated circulating proteins.** (**A**) Overview of proteome-wide two-sample MR testing the causal effect of obesity on 4,907 circulating proteins measured in deCODE. (**B**) Volcano plot summarizing proteome-wide MR results for obesity on circulating proteins. The x-axis shows the MR effect estimates (β_obesity), and the y-axis shows -log10(P values). Proteins passing the significance threshold are highlighted, with selected top 5 proteins labeled, including DNAJB12. (**C**) Scatter plots of SNP-specific effects illustrating the MR relationships between obesity and circulating DNAJB12. Each point represents an instrumental variant, and fitted lines correspond to IVW, weighted median, and MR-Egger estimates. (**D**) Scatter plots of comparison of MR effect estimates using obesity (OB) and BFP as exposures across circulating proteins. Each point represents one protein; proteins passing sensitivity tests are highlighted.

To ensure robustness and reduce false-positive findings, we applied a stringent post-MR filtering pipeline including multiple testing correction, heterogeneity assessment, horizontal pleiotropy evaluation, directionality testing, and reverse MR analyses. Following these procedures, 243 proteins remained as robust obesity-associated candidates, of which 35 showed decreased and 208 showed increased circulating levels in response to obesity (Supplementary Table 2), including DNAJB12 (*β*_OB-to-DNAJB12_ = 0.0922, *P* = 4.0210 × 10^−6^) (Fig. 2C).

Furthermore, to evaluate the consistency of obesity-related protein effects across different adiposity phenotypes, we integrated results from a two-step MR framework using BMI and BFP as alternative exposures with the same deCODE protein dataset as outcomes [10]. The MR effect estimates obtained using obesity as the exposure were highly correlated with those derived from BMI (*r*_OB-BMI_ = 0.902, *P* < 2.2 × 10^−16^) and BFP(*r*_OB-BFP_ = 0.836, *P* < 2.2 × 10^−16^) (Fig. 2D), confirming that these circulating proteins are causally regulated by adiposity. These results indicate that the identified 243 proteins represent systemic molecular consequences of adiposity and may serve as potential mediators linking obesity to downstream disease outcomes.

### 3.3 MR step 2: causal effect of circulating proteins on KOA

In the second step of the two-step MR framework, we assessed the causal effects of obesity-associated circulating proteins on the risk of KOA [20]. Specifically, we performed two-sample MR analyses for the 243 proteins identified in Step 1 using *cis*-pQTLs derived from the deCODE proteomics study as IVs (Supplementary Table 3). After this filtering step, 114 proteins with IVs ranging from 1 to 49 remained for MR analysis in Step 2 (Fig. 3A). Importantly, the *cis*-pQTL instruments used in Step 2 were distinct from the obesity-associated genetic instruments used in Step 1, with no overlap between the two sets of instrumental variables.

**Fig. 3:**
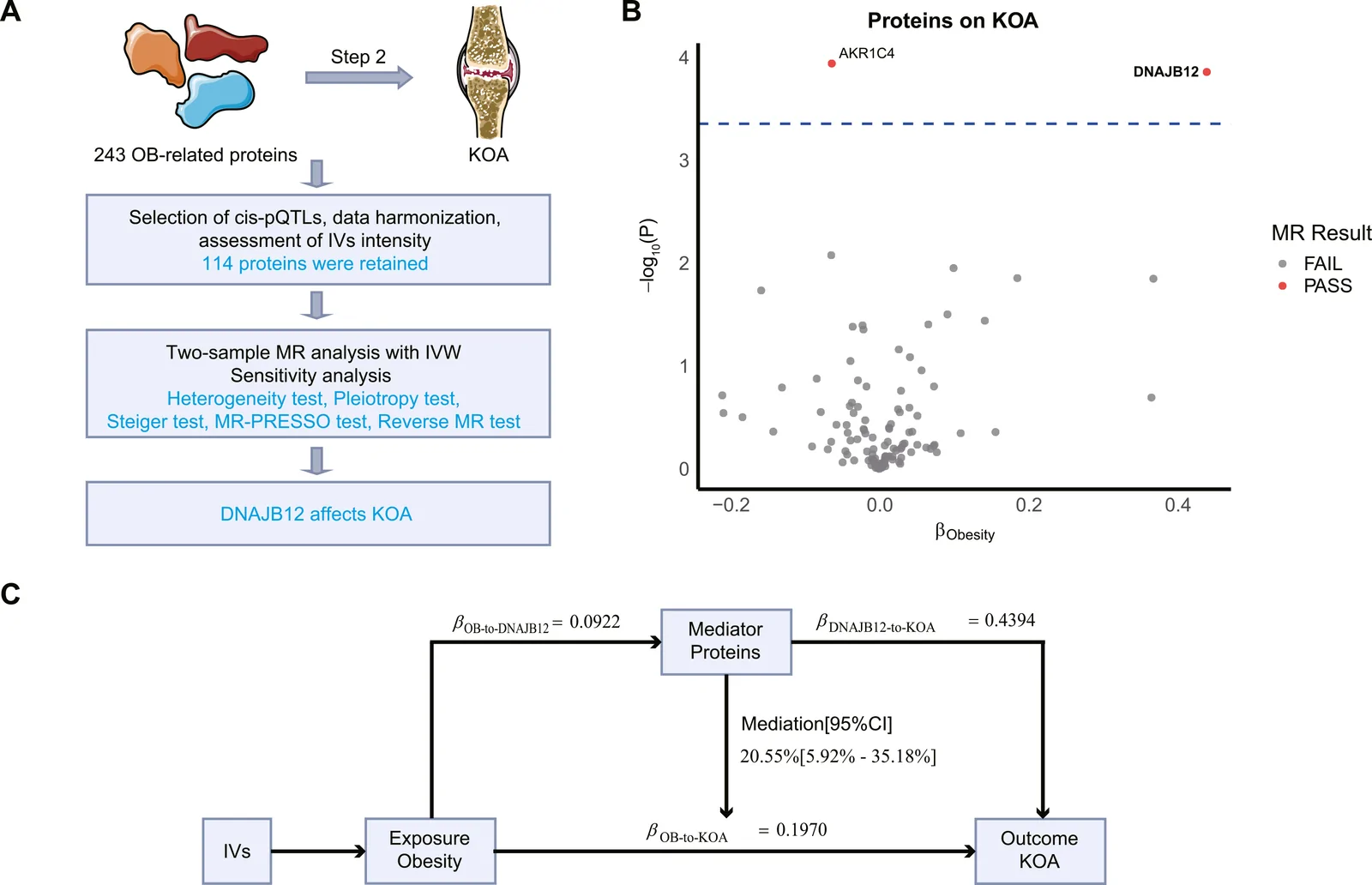
**Causal effects of obesity-regulated proteins on KOA and mediation by DNAJB12.** (**A**) Workflow of Step 2 in the two-step MR framework. *Cis*-acting pQTLs (*cis*-pQTLs) from the deCODE proteomics study were used as instrumental variables for obesity-related proteins identified in Step 1. After instrument selection, data harmonization, and sensitivity testing, 114 proteins were retained for MR analyses, and DNAJB12 were identified as having significant causal effects on KOA. (**B**) Volcano plot summarizing Step 2 MR results for the causal effects of obesity-related proteins on KOA. The x-axis indicates MR effect estimates, and the y-axis shows -log10(P values). (**C**) Schematic of mediation analysis using the product-of-coefficients approach. The total effect of obesity on KOA (*β*_0_) was decomposed into the indirect effect through mediator proteins (*β*_1_ × *β*_2_), and the mediating ratio was calculated as (*β*_1_ × *β*_2_/*β*_0_).

Following multiple testing correction and comprehensive sensitivity analyses including heterogeneity tests, pleiotropy assessments, and directionality evaluation, 2 proteins exhibited significant causal associations with KOA risk (Supplementary Table 4) (Fig. 3B). However, considering the consistency of the effect directions, only DNAJB12 was retained whose *β*_OB-to-proteins_ × *β*_proteins-to-KOA_ and *β*_OB-to-KOA_ had the same direction (Fig. 3C).

### 3.4 Mediation analyses

To estimate the roles of DNAJB12 in the OB-mediated risk of KOA, we calculated the mediating proportion using the product-of-coefficient method, and performed a Sobel test for sensitivity analysis (Fig. 3C). The IVW method confirmed that obesity significantly increases KOA risk (OR = 1.2178 (1.1596, 1.2789), *P* = 3.1377 × 10^−15^). We estimated that 20.55% (5.92%, 35.18%) of the effect of OB on KOA was mediated by circulating DNAJB12 (*P*_Sobel_ = 0.0059).

### 3.5 Replication MR for step 1 and step 2

To evaluate the robustness and generalizability of our findings, we conducted replication MR analyses using independent GWAS and pQTL datasets (Fig. 4A).

**Fig. 4:**
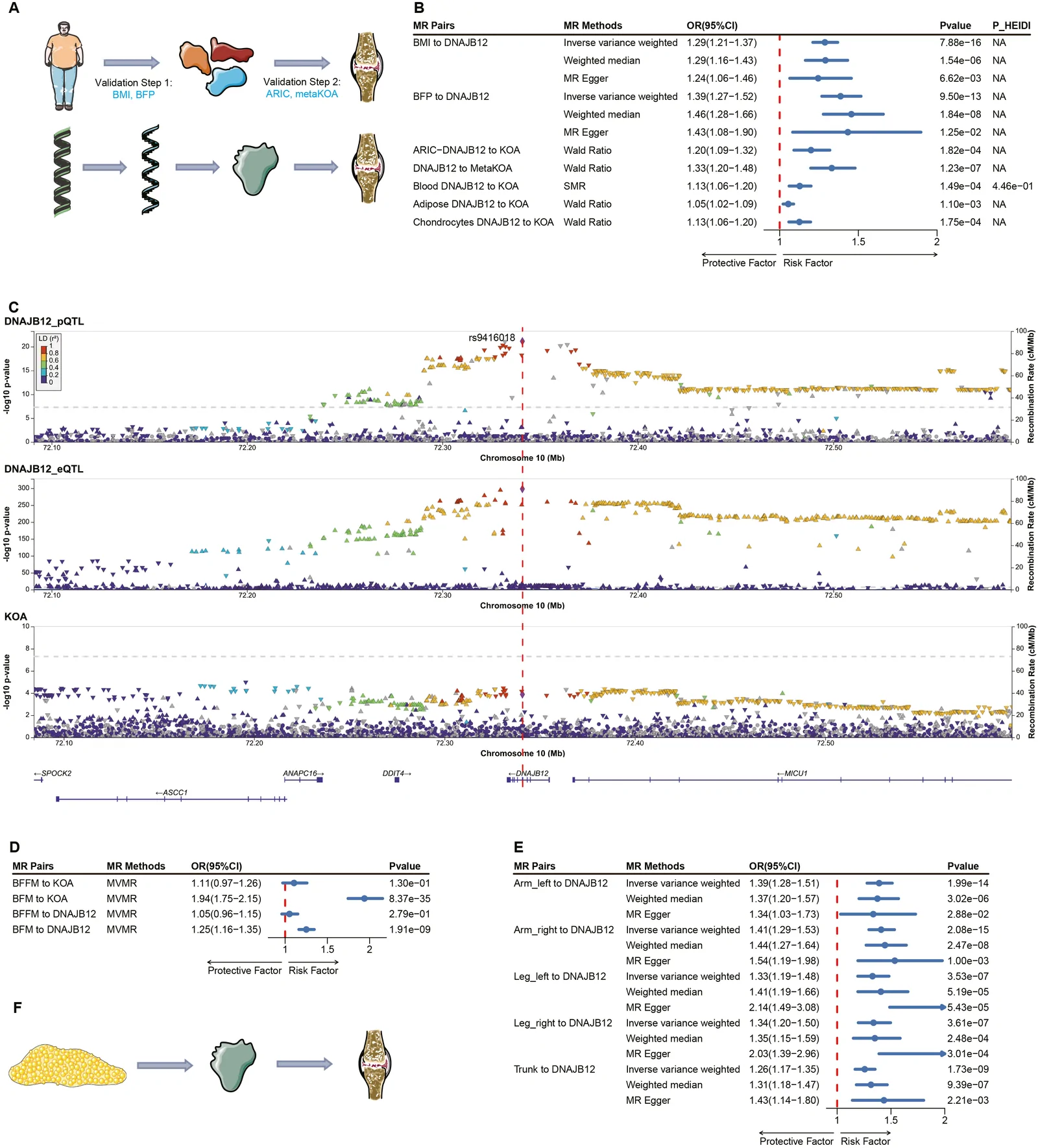
**Replication, transcript-level support, colocalization and body composition evidence for DNAJB12.** (**A**) Replication strategy integrating independent exposure GWAS and external pQTL/eQTL resources. (**B**) Replication MR using independent adiposity exposures (UK BMI; UK BFP), external *cis*-pQTL instruments from ARIC and external KOA outcome from the largest KOA meta GWAS summary data; transcript-level association assessed using summary-data-based MR (SMR) with HEIDI testing to exclude linkage-driven signals; adipose *cis*-eQTLs from METSIM study and chondrocyte *cis*-eQTLs from the United States. (**C**) Regional association plots at the DNAJB12 locus comparing *cis*-pQTL, *cis*-eQTL, and KOA GWAS signals, highlighting the lead variant rs9416018 and supporting shared genetic architecture via Bayesian colocalization (created using https://my.locuszoom.org/). (D) Multivariable MR analyses disentangling the independent effects of BFM and body fat-free mass on KNEE OA risk and on circulating DNAJB12 levels. Effect estimates are shown as ORs with 95% confidence intervals and corresponding P values. (E) Forest plot showing the influence of five body part fat percentages on DNAJB12. (F) Fat rather than muscle leads to an increase in DNAJB12 levels, thereby increasing the risk of KOA.

Because DNAJB12 has not been widely recognized as a canonical obesity-related protein, we further examined whether its association with obesity could be reproduced using alternative adiposity-related traits rather than relying solely on the binary obesity definition. For this replication analysis of Step 1, we used the BMI GWAS data [21] and a BFP GWAS data [22] as the exposure. Two-sample MR analyses were performed for DNAJB12. All associations remained statistically significant and passed sensitivity analyses, supporting the reproducibility of the causal effects of adiposity on these circulating protein levels (Fig. 4B) (Supplementary Table 5).

For step 2, we leveraged independent *cis*-pQTL dataset from the ARIC study [23] as alternative sources of protein instruments. The causal effects of DNAJB12 on KOA risk were consistently significant across datasets and remained robust in sensitivity testing (*β*_DNAJB12-to-KOA_ = 0.1816, *P* = 0.0002). At the same time, we utilized a dataset of the largest size to date but not purely of European origin to verify whether DNAJB12 increases the risk of KOA across different ethnic groups. The results show that DNAJB12 has a significant positive causal effect on the risk of KOA (OR = 1.3307 (1.1970, 1.4793), *P* = 1.2300 × 10^−7^) (Fig. 4B) (Supplementary Table 5).

### 3.6 Tissue-specific eQTL evidence for DNAJB12 in KOA

After establishing causal effects of circulating DNAJB12 protein levels on KOA risk, we further investigated whether the expression of this gene in blood exhibited consistent causal associations with KOA [25]. Demonstrating concordant evidence at the transcript level would support a mechanistic link spanning the full cascade from transcription to translation (Fig. 4A). We found that the gene expression levels of DNAJB12 (*β*_DNAJB12-SMR_ = 0.1199, *P*_DNAJB12-SMR_ = 0.0001) in the blood and tissues also increase the risk of KOA. The HEIDI test (*P*_DNAJB12-HEIDI_ = 0.4461) did not reveal any evidence of heterogeneity, suggesting that the association was not caused by LD, and supporting a potential causal relationship (Fig. 4B).

We next extended the analysis to adipose tissue and chondrocytes, which are more directly relevant to obesity and joint degeneration, respectively. Similar associations were observed using adipose tissue eQTLs (*β*_DNAJB12-adipose_ = 0.0532, *P*_DNAJB12-adipose_ = 0.0011) and chondrocyte eQTLs (*β*_DNAJB12-chondrocyte_ = 0.1180, *P*_DNAJB12-chondrocyte_ = 0.0002). Together, these results provide transcript-level support across blood, adipose tissue, and chondrocytes, suggesting that the DNAJB12–KOA association is not limited to circulating protein genetic instruments alone.

Furthermore, we queried the GTEx database to examine tissue-wide *cis*-eQTL signals for DNAJB12. The lead SNP that colocalized with KOA (rs9416018) was strong *cis*-eQTL across multiple tissues (29/34) (Supplementary Table 6), indicating that genetic regulation of circulating protein abundance may be partially mediated by mRNA level.

### 3.7 Colocalization of the *cis*-pQTLs and eQTLs with KOA

To assess whether the MR associations were supported by shared genetic signals, we performed Bayesian colocalization analyses [29]. For circulating DNAJB12 protein levels, *cis*-pQTL signals from deCODE and ARIC showed suggestive colocalization with KOA in the discovery GWAS dataset (PP·H4 = 0.7471 and 0.7490, respectively). In the larger KOA GWAS dataset, the deCODE DNAJB12 *cis*-pQTL signal showed strong colocalization with KOA risk (PP·H4 = 0.9490), supporting a shared causal variant at this locus. Tissue-specific eQTL colocalization showed moderate evidence in whole blood and chondrocytes (PP·H4 = 0.6883 and 0.6954, respectively), but weaker evidence in adipose tissue (PP·H4 = 0.4133). DNAJB12 blood eQTL and circulating pQTL signals showed very strong colocalization (PP·H4 = 0.9996), suggesting concordance between transcript-level and protein-level genetic regulation.

Collectively, these results indicate that the strongest colocalization evidence was observed at the circulating protein level, particularly in the larger KOA GWAS dataset. Although tissue-specific eQTL colocalization analyses did not provide high-confidence evidence, the moderate PP·H4 values observed in blood and chondrocytes still offer suggestive support for a transcript-level link between DNAJB12 regulation and KOA risk. (Fig. 4C) (Supplementary Table 7).

### 3.8 Body composition and regional adiposity analyses for circulating DNAJB12

To identify the body composition component driving the obesity–DNAJB12–KOA pathway, we performed MVMR including fat mass and fat-free mass simultaneously as exposures (Supplementary Table 8).

MVMR revealed that fat mass independently increased circulating levels of DNAJB12 (*β*_DNAJB12_ = 0.2237, *P* = 1.9074 × 10^−9^), while fat-free mass did not have these effects. Sensitivity analyses using multivariate MR-Egger analysis yielded consistent results without multiplicity (*P* = 0.2022 for multivariate MR-Egger intercept) (Fig. 4D).

We further evaluated the independent effects of fat mass and fat-free mass on KOA risk. Genetically predicted fat mass was significantly associated with an increased risk of KOA (OR = 1.9391 (1.7450, 2.1548), *P* = 8.3744 × 10^−35^). The intercept test indicated no evidence of multiplicity (*P* = 0.1462 for multivariate MR-Egger intercept) (Fig. 4D).

Regional adiposity analyses showed that genetically predicted fat percentage in the left arm, right arm, left leg, right leg and trunk was consistently associated with higher circulating DNAJB12 levels (Fig. 4E and F), suggesting that the DNAJB12 association reflects overall adiposity burden rather than a single fat compartment.

## 4 Discussion

In this study, we provide convergent genetic evidence supporting a causal effect of obesity on KOA and identify circulating DNAJB12 as a plausible mediator of this relationship. Although DNAJB12 is not a canonical obesity-related protein, its association with obesity was reproduced using multiple alternative adiposity-related traits, including BMI, body fat percentage, BFM, and regional fat percentage measures. Body composition analyses further suggested that the obesity-related increase in circulating DNAJB12 was driven primarily by fat mass rather than fat-free mass. Consistent associations across trunk and limb fat percentage traits indicate that DNAJB12 is more likely related to overall adiposity burden than to a single anatomical fat compartment. These findings strengthen the interpretation that DNAJB12 may represent an adiposity-responsive circulating protein linking obesity-related biological changes to KOA risk.

DNAJB12 encodes an endoplasmic reticulum (ER)-associated Hsp40 (DnaJ) co-chaperone that cooperates with Hsp70 to regulate protein folding and quality control at the ER membrane, including ER-associated degradation of misfolded proteins [32]. However, it was only found in proteomics and MR studies of OA that DNAJB12 increases the risk of OA [17,18]. Furthermore, studies on the role of DNAJB12 in other diseases have revealed that DNAJB12 can promote the progression of gastric cancer [33]. Although direct evidence linking DNAJB12 to obesity-related phenotypes remains limited, its biological role suggests that obesity-driven alterations in proteostasis pathways may influence circulating DNAJB12 levels and downstream disease risk. We speculate that endoplasmic reticulum stress and impaired protein balance may also be involved in the pathogenesis of osteoarthritis. This might be the key mechanism by which DNAJB12 mediates the effect of obesity on KOA.

As a co-activator protein of Hsp70, DNAJB12 has been verified to be able to regulate the ATPase and substrate binding activities of HSPA8/Hsc70 [[34], [35], [36]]. We speculate that the enhanced risk effect of DNAJB12 on KOA might be, to some extent, influenced by HSPA8/Hsc70. A study that combined bulk RNA sequencing and single-cell transcriptome sequencing revealed that HSPA8 is a potential diagnostic marker for OA [37]. HSPA8 is believed to be involved in the autophagy dysfunction that occurs during the progression of OA [38]. This all suggests that the biological functions mediated by HSPA8 and DNAJB12 may play a role in the progression of OA.

This study has several strengths, including large GWAS sample sizes for obesity and KOA, a proteome-wide two-step MR design to systematically identify candidate circulating mediators, and multiple layers of replication and sensitivity analyses to reduce the likelihood of false-positive findings. We additionally incorporated alternative obesity-related traits, body composition measures, regional adiposity phenotypes, independent pQTL datasets, tissue-specific eQTL resources, and Bayesian colocalization analyses to assess the consistency and biological plausibility of the DNAJB12 signal. However, several limitations should be acknowledged. MR estimates rely on instrumental variable assumptions, and residual horizontal pleiotropy cannot be fully excluded despite extensive sensitivity analyses. Circulating protein abundance may not fully capture tissue-specific protein activity or local biological processes within the joint. Although adipose tissue and chondrocyte eQTL analyses were included to improve tissue relevance, these datasets had smaller sample sizes than blood eQTL resources, limiting statistical power and colocalization resolution. The eQTL colocalization evidence was suggestive rather than definitive, particularly in adipose tissue, and therefore requires functional validation. Finally, the analyses were primarily conducted in European-ancestry populations, and the generalizability of these findings to other ancestries warrants further investigation.

In summary, our findings support a causal role of obesity in KOA and nominate circulating DNAJB12 as a genetically supported mediator linking adiposity burden to KOA risk. The results suggest that fat accumulation, rather than fat-free mass, is the major body composition component associated with increased circulating DNAJB12 levels. These findings provide mechanistic insight into obesity-associated KOA and suggest that strategies aimed at reducing adiposity may help modulate DNAJB12-related risk pathways, although experimental and clinical studies are needed to confirm whether lowering DNAJB12 or reducing adiposity can translate into reduced KOA risk.

## Contributions

SS conceived the overall theme of the paper, developed the method, and provided financial support. AW drafted the manuscript. AW, HS, YL, XB and DZ performed the data analysis. XH refined the method. XH and XC provided analytical suggestions. All authors contributed to writing the manuscript and approved the final version.

## Data availability

All data analysed in this study were obtained from publicly available summary-level GWAS, pQTL, and eQTL resources. The obesity GWAS summary statistics were obtained from FinnGen. Circulating proteome GWAS summary statistics were obtained from the deCODE and ARIC studies. The primary knee osteoarthritis GWAS summary statistics were obtained from the UK Biobank and arcOGEN meta-analysis, and the replication dataset was obtained from a large multi-ancestry osteoarthritis GWAS meta-analysis. Additional adiposity-related GWAS datasets, including BMI, body fat percentage, BFM, fat-free mass, and regional BFP traits, were obtained from GIANT, UK Biobank, IEU OpenGWAS, and the GWAS Catalog. *Cis*-eQTL summary statistics were obtained from eQTLGen, METSIM, GTEx, and publicly available chondrocyte eQTL resources. Detailed information on all datasets, including accession IDs, sample sizes, ancestry information, and URLs, is provided in Supplementary Table 1. All results are included in this article and its supplementary information files.

## Code availability

The all original scripts used in this study are publicly available at: https://sqsun.github.io/software.html.

## Ethics declarations

We obtained genotype, protein and phenotype data from the UKB and UKB-PPP, which received ethics approval from the North West Centre for Research Ethics Committee. The application number of UKB and UKB-PPP in our study was 88159 and 67665.

## Declaration of generative AI in scientific writing

No artificial intelligence tools were used in the preparation of this manuscript.

## Funding

This study was supported by the National Natural Science Foundation of China (Grant No. 82574212); the Fundamental Research Funds for the Central Universities, Xi'an Jiaotong University (Grant Nos. xtr052024011, 11913222000003, 11913222000007, and xzy012025154); the Natural Science Basic Research Program of Shaanxi (Grant Nos. 2024JC-YBMS-575, 2023-JC-QN-0737 and 2025JC-YBQN-1044); Shaanxi Postdoctoral Research Program (Grant No. 2024BSHSDZZ046).

## Declaration of competing interests

The authors declare no conflict of interests.
